# Patients’ attitudes regarding characteristics of physicians in ophthalmology

**DOI:** 10.1186/s13104-017-3056-0

**Published:** 2017-12-11

**Authors:** Lauren Mason, John Mason

**Affiliations:** 1 Retina Consultants of Alabama P.C., 700 18th Street South, Suite 707, Birmingham, AL 35233 USA; 2 Department of Ophthalmology, University of Alabama-Birmingham, Birmingham, AL USA

**Keywords:** Patient survey, Vitreoretinal specialist appearance, Vitreoretinal specialist attire, Vitreoretinal specialist education

## Abstract

**Objective:**

This retrospective cross-sectional study was performed to assess patient perceptions and attitudes towards physicians’ physical appearance and education in the Vitreoretinal Specialist clinic setting. 295 consecutive patients from Retina Consultants of Alabama, a Vitreoretinal Specialty practice at UAB Hospital, completed a questionnaire assessing preferences regarding physicians’ appearance and education. The main outcome measure was to determine if physical attributes and education are factors in patient preference for their Vitreoretinal Specialist.

**Results:**

There were no significant desirable or undesirable physical characteristics for a Vitreoretinal Specialist in a clinical setting. However, the data indicated trends in patient preferences for Vitreoretinal Specialist attire [209 (71%) of 295 patients prefer casual dress], physical appearance [212 (72%) and 240 (81%) of 295 patients had no preference with regards to long hair and facial hair], and medical education [171 (58%) of 298 patients preferred their Specialist to be involved in research and education]. Gender, race, and age were not significantly associated with patients’ perceptions toward the Vitreoretinal Specialist’s physical appearance and education. In conclusion, Vitreoretinal Specialist’s clothing and physical attributes do not significantly influence patients’ opinion of the care they receive, although patients prefer their Specialist to be involved in research and continuing education.

## Introduction

The role of a physician in any facet of medicine is to effectively prevent and treat disease by making the patient needs a priority. Research has shown that a patient’s initial encounter with a medical care provider is essential for establishing the communication and trust crucial to providing optimal care [1]. Studies have shown a strong correlation between physician appearance and patient’ initial perceptions of physician competence [2–4].

Furthermore, societal norms and traditions have paved the foundation for shaping the way that patients view their physicians’ appearance in the workplace. Physician dress and the impact it has on the physician–patient relationship can be traced back to Hippocrates, who claimed that the physician “must be clean in person, well dressed, and anointed with sweet smelling unguents…” [5] Studies from two decades ago found that patients preferred and gave more positive feedback towards traditional physician attire, including a white coat, nametag, and business casual attire [6]. However, the preferred public image of how physicians in the United States should look may have changed in recent years with the evolution in technology, medicine, and American culture.

Although previous studies have looked at patient preference on physician appearance in several different areas of medicine such as primary care and dermatology [6, 7], this is the first study to apply this data to the field of Vitreoretinal disease and surgery. The purpose of our study was to assess patient perceptions with regard to physician appearance and determine whether physician appearance is significantly associated with overall patient satisfaction.

## Main text

### Methodology

Institutional Review Board (IRB) approval and informed consent was obtained prior to collection of patient data from the University of Alabama-Birmingham IRB. 295 consecutive patients participated in a cross-sectional survey assessing preferences for desirable and undesirable characteristics for their Vitreoretinal Specialist. The study took place over 9 months, from March to December 2015, at Retina Consultants of Alabama at UAB Hospital. Each patient completed the written survey in the physician’s office prior to the examination. No physicians or investigators were present while the survey was administered in the waiting room. No patients were excluded. Sample size was determined by the UAB statistical department using 95% confidence level and a standard deviation of 0.5. Gender, race, and age were the demographic traits taken into account.

Based on previous literature regarding patient preferences for their physician [1–7], a cross sectional written survey was created at Retina Consultants of Alabama. Participants were asked a series of nine questions, each comprising a different physical attribute associated with physician appearance. Each question assessed which characteristics patients prefer in a physician with regards to attire, physical features, age, and medical training.

For attire, participants reported that characteristics were desirable, undesirable, or no preference. Patient preference for traditional white coat or casual dress, preference for dress shoes or casual shoes, and presence of a nametag were recorded. Negative patient views of physical features such as long hair, facial hair, and visible tattoo or piercings was also evaluated, as well as a preferred physician age range. Patients were asked if they had a preference for where the physician did their medical training, and if the physician is involved in medical research. Each question was selected because of its use in previous research evaluating patient preference for physician characteristics and appearance.

A total of 295 patients completed the survey (Table 1) for patient preference regarding Vitreoretinal Specialist appearance. 134 males and 161 females participated in the study. Of the 295 patients, 228 were Caucasian, 64 were African American, and 3 were Native American. Mean patient age was 66 (range 55–75).

**Table 1:** The questionnaire

Questions	
1. What attire do you prefer your Ophthalmologist to wear?	
a. White coat b. Formal suit and tie c. Semiformal d. Casual e. Scrubs f. Jeans g. No preference	
2. What shoes do you prefer your Ophthalmologist to wear?	
a. Dress shoes b. Sneakers c. Clogs d. Sandals e. No preference	
3. Do you prefer a nametag?	
4. Do you have a negative view of long hair?	
5. Do you have a negative view of facial hair?	
6. Do you have a negative view of visible tattoos or body piercings?	
7. Do you have a preferred age range?	
a. 30–39 b. 40–49 c. 50–59 d. 60–69 e. No preference	
8. Do you care where your Ophthalmologist did their medical training?	
9. Do you care if your Ophthalmologist is involved in medical research?	

Chi square analysis was performed to determine if a correlation exists between patients’ demographic characteristics (gender, race, and age) and patient preferences for physician appearance. Cross tabulation analysis of questionnaire responses revealed that patient demographics do not independently contribute to statistically significant patient preferences with regard to attire, physical characteristics, or training associated with their Vitreoretinal Specialist.

### Results

Patients’ exhibited variability regarding their Vitreoretinal Specialist’s attire. Only 83 (28%) of 295 patients preferred a traditional white coat, while an overwhelming 210 (71%) of 295 patients preferred casual dress, scrubs, jeans, or had no preference for physician attire. There was no significant correlation between patient demographics (gender and race) and preference for Vitreoretinal Specialist’s attire (p = 0.60, p = 0.13) (Table 2). 207 (70%) of 295 patients showed no shoe preference for their Vitreoretinal Specialist, while 230 (78%) of 295 patients prefer their Vitreoretinal Specialist to wear a nametag.

**Table 2:** Demographic features of responding patients (gender and race)

	Gender (Males n = 134; Females n = 161)		p value
	Male	Female	
Q-1 (prefer traditional white coat)			0.599
Yes	39 (29%)	43 (27%)	
No	89 (67%)	106 (67%)	
No preference	5 (4%)	10 (6%)	
Q-2 (prefer dress shoes)			0.487
Yes	28 (21%)	32 (20%)	
No	15 (11%)	15 (9%)	
No preference	91 (68%)	114 (71%)	
Q-3 (prefer nametag)			0.584
Yes	103 (77%)	128 (80%)	
No	31 (23%)	33 (20%)	
Q-4 (negative view of long hair)			0.13
Yes	44 (33%)	40 (25%)	
No	90 (67%)	121 (75%)	
Q-5 (negative view of facial hair)			0.792
Yes	25 (19%)	32 (20%)	
No	109 (81%)	129 (80%)	
Q-6 (negative view of tattoos/piercings)			0.521
Yes	80 (60%)	102 (63%)	
No	54 (40%)	59 (37%)	
Q-7 (preferred age range)			0.953
Yes	33 (25%)	39 (24%)	
No	101 (75%)	122 (76%)	
Q-8 (preference where MD trained)			0.566
Yes	53 (40%)	69 (43%)	
No	81 (60%)	92 (57%)	
Q-9 (preference if MD does research)			0.726
Yes	77 (57%)	94 (58%)	
No	2 (1%)	4 (2%)	
No preference	55 (41%)	62 (40%)	

Race	Caucasian	African American	Native American	p value
Q-1 (prefer traditional white coat)				0.1337
Yes	55 (25%)	26 (41%)	1 (33.3%)	
No	159 (70%)	35 (56%)	1 (33.3%)	
No preference	12 (5%)	2 (3%)	1 (33.3%)	
Q-2 (prefer dress shoes)				0.799
Yes	44 (19%)	15 (23%)	1 (33.3%)	
No	23 (10%)	6 (10%)	1 (33.3%)	
No preference	161 (71%)	43 (67%)	1 (33.3%)	
Q-3 (prefer nametag)				0.0578
Yes	172 (75%)	57 (89%)	2 (66.7%)	
No	56 (25%)	7 (11%)	1 (33.3%)	
Q-4 (negative view of long hair)				0.981
Yes	65 (29%)	18 (28%)	1 (33.3%)	
No	163 (71%)	46 (72%)	2 (66.7%)	
Q-5 (negative view of facial hair)				0.101
Yes	38 (17%)	18 (28%)	1 (33.3%)	
No	190 (83%)	46 (72%)	2 (66.7%)	
Q-6 (negative view of tattoos/piercings)				0.094
Yes	148 (65%)	32 (50%)	2 (66.7%)	
No	80 (35%)	32 (50%)	1 (33.3%)	
Q-7 (preferred age range)				0.117
Yes	52 (23%)	19 (30%)	1 (33.3%)	
No	176 (77%)	45 (70%)	2 (66.7%)	
Q-8 (preference where MD trained)				0.583
Yes	91 (40%)	30 (47%)	1 (33.3%)	
No	137 (60%)	34 (53%)	2 (66.7%)	
Q-9 (preference if MD does research)				0.579
Yes	129 (57%)	40 (63%)	2 (66.7%)	
No	5 (2%)	1 (2%)	0 (0%)	
No preference	94 (41%)	23 (36%)	1 (33.3%)	

The actual number represents each participant’s response to the question, while the parenthesis gives the percentage of responses to the question

Physical attributes such as hair length, facial hair, and visible tattoos and body piercings were included in the questionnaire. 212 (72%) of 295 patients did not have negative views associated with long hair in their physician. However, men were more likely to be offended by longer hair than women were, although not statistically significant (p = 0.13). Race showed no effect on patient preference for Vitreoretinal Specialist hair length (p = 0.98). With regards to facial hair, 239 (81%) of 295 patients did not have negative views associated with physician facial hair. Demographic factors (gender and race) did not play a role in determining preference for Vitreoretinal Specialist facial hair (p = 0.79, p = 0.1). With regards to visible tattoos and piercings, 183 (62%) of 295 patients reported that they did not have negative views associated with visible body markings and piercings. However, demographic factors (gender and race) played no substantial role in determining patient views of their Vitreoretinal Specialist having tattoos and piercings (p = 0.52, p = 0.09). Furthermore, slightly younger patients were overall more accepting towards long hair and visible tattoos and piercings compared to those who were older (60 years old vs 70 years old), although not statistically significant.

As it pertains to medical training and furthering education of their Vitreoretinal Specialist, 174 (59%) of 295 patients reported having no preference where the physician did their medical training. Demographic factors (gender and race) had no significant effect on patient preference of their Vitreoretinal Specialists training (p = 0.566, p = 0.583). 171 (58%) of 295 patients prefer their medical care provider to be involved in current medical research (Table 3). There was no significant correlation between demographic factors (gender and race) and patient preference for their Vitreoretinal Specialist’s continued education (p = 0.73, p = 0.58).

**Table 3:** Responses to survey questions

Males n = 134; Females n = 161	Yes	No	No preference
Q-1 (prefer traditional white coat)	82 (28%)	195 (67%)	15 (5%)
Q-2 (prefer dress shoes)	60 (20%)	30 (10%)	205 (70%)
Q-3 (prefer nametag)	231 (78%)	64 (22%)	0
Q-4 (negative view of long hair)	84 (28%)	211 (72%)	0
Q-5 (negative view of facial hair)	57 (19%)	238 (81%)	0
Q-6 (negative view of visible tattoos/piercings)	182 (62%)	113 (38%)	0
Q-7 (preferred age range)	72 (24%)	223 (76%)	0
Q-8 (preference where MD trained)	122 (41%)	173 (59%)	0
Q-9 (preference if MD does research)	171 (58%)	6 (2%)	118 (40%)

The actual number represents each participant’s response to the question, while the parenthesis gives the percentage of responses to the question

### Discussion

Regarding attire, in our study only 83 (28%) of 295 patients preferred a traditional white coat, while an overwhelming 210 (71%) of 295 patients preferred casual dress, scrubs, jeans, or had no preference for physician attire. This finding is important because previous studies in different areas of medicine have shown that patients prefer their physician to wear a traditional white coat. Our study differs in that Vitreoretinal Specialists are not preferred by patients to wear a white coat. For example, Keenum et al. found that in a family practice setting, patients prefer a traditionally dressed physician as opposed to a physician who is dressed casually. Their findings suggest that a family practice provider wear a nametag, white coat, and should avoid wearing clogs, sandals, and jeans in order to obtain highest patient care [6]. Kanzler et al. found similar results in a dermatologic practice setting, claiming dermatologists should wear a name badge, white coat, and dress shoes in the clinical setting when consulting with patients. Casual dress, jeans, scrubs, and tennis shoes were noted to be undesirable characteristics found by patients, and are to be avoided in the clinic setting [7]. In an internal medicine setting, Rehman et al. found that respondents overwhelmingly favor physicians in professional attire with a white coat (76.3%, p = 0.0001). Patients reported that they were significantly more willing to share social, sexual, and psychological problems with the physician who is professionally dressed (p = 0.0001), suggesting that wearing professional dress while consulting with a patient may favorable influence trust and confidence building [8]. Our results reveal that the majority of patients showed no preference regarding traditional physician attire, white coat, or certain physical characteristics for their Vitreoretinal Specialist in a Vitreoretinal practice setting. These results compare favorably with a surgical study done by Edwards et al. that suggests that surgeons’ clothing does not significantly influence patient’s opinion of the care they receive, and that patients do not have a preference for white coats or more traditional surgical attire [9].

As it pertains to physical appearance, including hair length, facial hair, and tattoos and piercings, there was no statistically significant effect on patient preference and perception of their Vitreoretinal Specialist with regards to these characteristics. Facial hair did not have an effect on patient’s perception, as 236 (80%) of 295 patients reported having no negative views of Vitreoretinal Specialists with facial hair, while 212 (72%) of 295 patients did not report negative feelings towards their Vitreoretinal Specialist having long hair. This finding is markedly different from previous literature in the field of dermatology. Kanzler et al. found that in a dermatologic practice, to best serve their patients, long hair should be avoided in the clinical setting during a consultation [7]. This suggests that in a Vitreoretinal Specialist setting, patients are more focused on the quality of their eye care, rather than a physical characteristic of their physician. With regards to tattoos and piercings, previous studies have shown that tattoos and piercings adversely affect confidence rates of patients. Johnson et al. reported that visible tattoos and piercings on a medical care provider directly affect patients’ perception and trust of the provider’s capabilities. Tattooed physicians were given lower confidence ratings by patients, while patients reported greater levels of discomfort around physicians with visible facial piercings [10]. This does not support the results of our study, which showed that 183 (62%) of 295 patients did not report a negative view of a Vitreoretinal Specialist with visible tattoos and piercings.

With regards to medical training and furthering education of their Vitreoretinal Specialist, 175 (59%) of 295 patients reported having no preference where the physician did their medical training, whereas 171 (58%) of 295 patients prefer their Vitreoretinal Specialist to be involved in current medical research. Abghari et al. reported no significant correlation between patient demographics and importance placed on the educational background of their physician in an orthopedic clinic setting. The study confirmed that patient perceptions of their orthopedic surgeon seem to be based exclusively on the quality of education and experience of their physician [11]. Farquharson et al. highlights the importance of the continued education of a physician and the value of participation in medical research. Continued education through research fosters lessons in teamwork, respect for routine, sharpens judgement, and can ultimately cultivate appreciation for a crucial aspect of medicine necessary for the advancement of healthcare [12]. Our Vitreoretinal study results confirm these trends in patient preference for research involvement and continued education for surgeons.

### Conclusions

This is the first study to evaluate the significance between Vitreoretinal Specialist attributes and patient preference in the field of Vitreoretinal disease and surgery. Attire and physical characteristics do not significantly influence a patient’s opinion of their Vitreoretinal Specialist. Patients do not have a strong preference for white coats and traditional clinical attire, as the majority of patients have no preference for what their Vitreoretinal Specialist is wearing. With regards to medical training, patients do not show a preference for where their Vitreoretinal Specialist trained. However, data shows that patients value the Vitreoretinal Specialist who participates in continuing education and research. Additional patient surveys in the field of subspecialty surgery may further define patient preferences for their surgical subspecialist.

## Limitations

There are several limitations in our study consistent with a survey of patients and the potential bias inherent in a survey. We cannot exclude that the potential lack of statistical significance in some aspects of the questionnaire may be due to insufficient participants required to find statistical power. We did not evaluate level of education and income, and 227 (77%) of 295 patients surveyed within our sample were Caucasian. Our study may have differed from previous studies with regards to patient demographics, as well as regional preferences. However, we believe that our large sample size can lead to statistically valid conclusions.

## Abbreviation

IRB: Institutional Review Board
